# Prediction of Ultimate Capacity of Concrete Columns Reinforced with FRP Bars

**DOI:** 10.3390/polym15051161

**Published:** 2023-02-25

**Authors:** Jacek Korentz, Witold Czarnecki

**Affiliations:** Institute of Civil Engineering, University of Zielona Góra, Prof. Z. Szafrana 1, 65-516 Zielona Góra, Poland

**Keywords:** load-bearing capacity, RC column, FRP bars, axial force-bending moment interaction, design

## Abstract

FRP bars are used in concrete structures as an alternative to steel bars as they have many advantages such as high tensile strength, high strength-to-weight ratio, electromagnetic neutrality, lightweight and no corrosion. There is a perceived lack of standard regulations for the design of concrete columns with FRP reinforcement, e.g., in Eurocode 2. This paper describes a procedure for predicting the bearing capacity of concrete columns with FRP reinforcement based on the interaction of axial force and bending moment, which was developed on the basis of existing design recommendations and standards. It was shown that the bearing capacity of eccentrically loaded RC sections depends on two parameters, which are the mechanical reinforcement ratio ω and the location of the reinforcement in the cross-section expressed by the β factor. The analyses carried out showed the existence of a singularity in the n–m interaction curve indicating the fact that in a certain loaded range, the curve is concave, and more it was shown that the balance failure point for sections with FRP reinforcement takes place for eccentric tension. A simple procedure for calculating the required reinforcement from any FRP bars in concrete columns was also proposed. Nomograms developed from n–m interaction curves provide for the accurate and rational design of FRP reinforcement in columns.

## 1. Introduction

Reinforced concrete structures are often exposed to destructive environmental influences such as the effects of moisture, salt, frost, acid and frequent changes in temperature and loads. These are factors that cause accelerated corrosion of the steel reinforcement in RC elements. For this reason, non-metallic reinforcement made of FRP (Fibre Reinforced Polymer) bars is increasingly used in concrete structures. The market offers a wide range of FRP bars made from different materials with very different mechanical properties. Composite bars have a very high tensile strength, and, in most cases, a low modulus of elasticity compared to steel bars. The tensile properties of FRP bars have linear elastic characteristics up to rupture. Due to the lack of plastic deformation, the failure occurs suddenly. Therefore, the behaviour of concrete columns reinforced with FRP bars is different from that of columns reinforced with steel bars.

A complete overview of the approach and design philosophy of concrete structures reinforced with FRP composites is presented in Fib Bulletin [1]. In the design of concrete structures with FRP reinforcement, either the guidelines [2] or a few national standards, among others [3,4,5,6], can be used. An overview of the design recommendations and standards for the design of concrete elements reinforced with FRP bars are presented in the works [7,8].

However, due to the very conservative provisions of some standards [3,4] and the lack of standard regulations in the European standards, i.e., in Eurocode 2 [9], concerning the design of concrete columns with FRP reinforcement, further experimental research in this field is necessary as well as proposals for analytical methods to properly capture and present the performance of concrete columns with such reinforcement.

Numerous formulas for calculating the capacity of axially compressed columns can be found in the literature [10], but there are few for the case of eccentric compression [11]. Currently, the literature on the subject lacks any practical analytical formulas for the behaviour evaluation and design of concrete columns with FRP bars. However, commercial programs can be used to determine the design M–N interaction curves.

Recommendations [1,2] only concern the bending and shear resistance of RC beams. Therefore, this article fills an important gap in this area. This paper evaluates the performance of eccentrically loaded columns with FRP reinforcement based on the interaction between axial force and bending moment. For this aim, formulas were developed to determine the coordinates of the characteristic points of the interaction curve, considering the current design recommendations and standards for the design principles of concrete structures with FRP reinforcement. For a broad and comprehensive analysis, the nominal axial and flexural resistance were calculated in two scenarios: neglecting and considering the contribution of FRP bars in compression. Based on the formulas and diagrams developed, a simple procedure for the design of reinforcement from any FRP bars in concrete columns was proposed.

## 2. Properties of FRP Reinforcement

FRP bars are made of continuous fibres of various high-strength materials and a matrix, which is a polymer resin. The strength characteristics of composite bars depend, among other things, on the type of fibre used, the type of matrix and the fibre saturation of the matrix. Rods made from FRP are characterized by, among other things, high tensile strength, corrosion resistance and a lack of magnetic properties. The most commonly used materials for non-metallic bars are glass fibre (GFRP), carbon fibre (CFRP), basalt fibre (BFRP) and aramid fibre (AFRP).

The mechanical properties of FRP reinforcement are characterized by three parameters: tensile strength *f_f_*, ultimate strain *ε_fu_* and elastic modulus *E_f_*. These parameters vary widely and depend primarily on the type of fibre. Thus, for CFRP bars: f_f_ = 600–3000 MPa, *ε_fu_* = 0.5–1.8%, *E_f_* = 80–500 GPa, for GFRP bars: *f_f_* = 400–1600 MPa, *ε_fu_* = 1.2–3.7%, *E_f_* = 30–60 GPa, for AFRP bars: *f_f_* = 600–2500 MPa, *ε_fu_* = 1.8–4.0%, *E_f_* = 30–125 GPa [12] and for BFRP bars: *f_f_* = 1100–1450 MPa, *ε_fu_* = 2.2%, *E_f_* = 55–78 GPa. In order to improve the mechanical properties of FRP bars and reduce their manufacturing costs, research is being conducted on bars made of Hybrid Fibre-Reinforced Polymer (HFRP), in which carbon and basalt fibres and various resins are applied [13].

The nature of FRP bars confers them orthotropic properties. Therefore, their compressive strength is different from their tensile strength, where the compressive strength is much lower. For GFRP and CFRP bars, the compressive strength is between 30% and 60% of the tensile strength, depending on the diameter of the bars [14]. However, the compressive elastic modulus of GFRP bars is close to the tensile elastic modulus [15]. In addition, some composite bars prove to be sensitive to environmental influences, e.g., high alkalinity, UV exposure, elevated or reduced temperature, cyclic wetting and drying and cyclic freezing [8]. They are also characterised by low resistance to creep under long-term loads [16] and require appropriate technological treatments to ensure adequate bonding with concrete [17]. Therefore, the design values of FRP reinforcement paraments are much smaller than their characteristic values. They are determined according to the rules [18], in which the partial material factor and the relevant conversion factors are introduced.

## 3. Performance of Columns Reinforced with FRP Bars

Numerous experimental studies on the behaviour of mainly short concrete columns reinforced with GFRP, CFRP and BFRP bars under axial and eccentric forces have been carried out [19,20,21,22,23,24,25]. Numerical analyses are also being carried out [26,27,28] and analytical formulas are being developed for the design of FRP reinforcement [25,28,29].

Experimental results [21] show that the axial load-carrying capacity of columns of rectangular cross-section with CFRP bars is comparable to that of columns with steel reinforcement with the same ratio of reinforcement, and for eccentrically loaded columns, their bearing capacity is up to 12% lower depending on the eccentricity of the axial force. For eccentrically loaded columns (e/h = 1/3) with GFRP bars, the decrease in bearing capacity is greater; the bearing capacity is approximately 30% lower than that of columns with steel bars. The bearing capacity of rectangular columns axially and eccentrically loaded with GFRP and CFRP bars is strongly influenced by the spacing of the ties and their configuration; decreasing the tie spacing increases the bearing capacity of the columns by 10% to 20% [22,24]. Tests [20] of eccentrically loaded columns (e/D ≤ 1/3) with circular cross-sections reinforced with CFRP bars showed that their bearing capacity was 4% lower than that of their counterparts with steel reinforcement and was 2% higher for the axial force eccentricity e/D = 2/3. In contrast, axially compressed columns of circular cross-section with CFRP bars showed bearing capacities up to 12% higher than the reference columns with steel reinforcement [19]. In all the studies cited, the mechanism for the exhaustion of the load capacity of composite reinforced columns was the same. First, there is crushing and spalling of the concrete cover, followed by buckling in the longitudinal bars and crushing in the concrete in the core of the section, accompanied by large deformations. It should be mentioned that buckling in the reinforcement bars in the columns can be accelerated by geometrical imperfections of these bars caused by transverse deformations of the concrete [30].

In order to improve the effectiveness of FRP reinforcement, there are also studies of concrete columns with hybrid reinforcement, e.g., steel bars and GFRP bars [31], as well as studies of columns with fibre concrete and CFRP or GFRP bars [32,33], which show that such solutions are available.

The experimental results of columns with FRP reinforcement presented above correspond to the results of FEM analyses [19], which found a strong influence of the axial force eccentricity and the type of FRP bars on the bearing capacity of the sections. When the axial force eccentricity is e/d ≤ 0.5, the bearing capacity of columns with GFRP bars is several per cent lower than that of columns with steel reinforcement, while an increase in bearing capacity of up to several per cent is observed when CFRP bars are used. Other experimental studies also agree with the results of FEM analyses [28].

To avoid over deformation of elements reinforced with FRP bars, the limit strains and limit stresses in tension bars are reduced to ε_f_ = min (ε_fu_; 0.010) and f_f_ = min (f_fu_; 0.010 E_f_), respectively [34], and the stress in the compression bars is limited to 40% of the tensile strength [20]. Standards [3,4] prescribe the neglect of FRP compression bars in the analysis pf the bearing capacity of a column. However, studies [35,36] indicate that the skip of the strength of the FRP compression bars is too conservative since the tested bearing capacity of the axially compressed columns is higher than that derived from the standard estimates. Similarly, according to studies [23], the contribution of longitudinal FRP reinforcement should not be neglected in the calculation of the resistance of axially loaded columns, since the results of analytical solutions based on elastic theory give good agreement with experimental data.

In summary, it can be concluded that the experimental results to date and some numerical and analytical analyses indicate some discrepancies with the standards in estimating the resistance of columns with FRP bars. Therefore, further research is needed. In order to make the right choice for the type of FRP reinforcement, a method of predicting and evaluating the behaviour of columns reinforced with such bars over the entire load range is needed. This can be achieved by analysing the axial force-bending moment interaction curves for different FRP reinforcements.

## 4. Ultimate Capacity of RC Columns

According to the standards [3,4], for each applicable factored load combination in columns, the design strength at all sections shall satisfy and interaction between load effects shall be considered. In a study [25], a theoretical model was developed to predict the axial force-bending moment interaction diagrams in circular section columns reinforced with BFRP and GFRP bars. The principles of equilibrium and strain compatibility were used to develop the theoretical diagrams. In [28], the axial compression behaviour of hollow core concrete columns reinforced with GFRP was investigated numerically and empirically. Based on the data base obtained from ABAQUS and the laboratory test, various empirical formulas were proposed to predict the axial compression resistance of columns. In [29], an analytical diagram of the interaction between axial load and bending moment was developed for square section columns reinforced with GFRP bars. The small strip concrete method was used to determine the response of the compression concrete.

**Figure 1:**
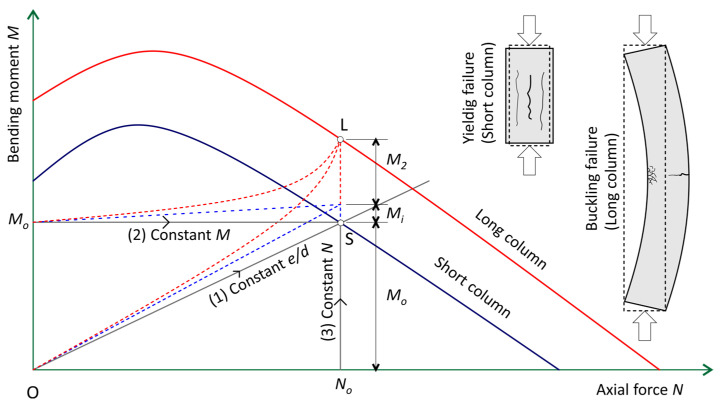
Interaction curves and load paths.

The strength of an eccentrically loaded RC section is equal to the values of the axial force N and the bending moment M lying on the interaction curve N–M (Figure 1) determined by the allowable strain of the concrete and the longitudinal reinforcement. The ultimate axial force N_u_ and the ultimate bending moment M_u_ for a specified state of stress are determined from the relation:

(1) $$N_{u}=\iint \sigma_{c}dA_{c}\pm \sigma_{f1}A_{f1}\pm \sigma_{f2}A_{f2}$$

(2) $$M_{u}=\iint \sigma_{c}z_{c}dA_{c}\pm \sigma_{f1}S_{f1}\pm \sigma_{f2}S_{f2}$$

where: A_c_ is the cross-sectional area of the compressed zone of the concrete, σ_c_ is the compressive stress distribution in the concrete, σ_f1_ and σ_f2_ are the stresses in the tension and compression bars, respectively, A_f1_ and A_f2_ are cross-sectional area of the tension and compression bars, respectively, S_f1_ and S_f2_ are the static moments of the cross-sectional area of the tension and compression bars, respectively, related to the centre of gravity of the section.

The bearing capacity of a reinforced concrete column depends primarily on its slenderness [37]. If the column is short, there is material failure. If the column is slender, failure occurs through buckling at a bearing significantly lower than the strength of the materials used. There is another known mode of shear failure of slender columns [38], which occurs primarily during seismic loads.

There are often many load combinations in columns, with three static equilibrium paths possible [39], as illustrated in Figure 1: (1) Axial force acting on a constant eccentric. This is the case for loads transferring to the column from prefabricated beams. (2) Constant bending moment and increasing axial force. Such a situation may result in multistorey building columns in which moments are primarily due to adjacent floor loading. (3) Constant axial force but increasing bending moment. Such a situation may result, in an approximate manner, in building columns or in bridge piers, when they are subjected to lateral wind loads that seldom modify the column axial load but considerably increase the column bending moments. The equilibrium paths are different for short columns—OS path and slender columns—OL path.

For the design of a column to be considered adequate (safe), the combination of action effects (M, N) must be less than the combination of design strengths (M_u_, N_u_) from the interaction curve.

For slender columns, it is necessary to consider the effect of geometric imperfections with an additional bending moment M_i_ and the impact of second-order effects expressed by bending moment M_2_. The N–M interaction curves are very useful in the analysis of the bearing capacity of concrete columns with any reinforcement for all possible load paths. To simplify the analysis, the N–M interaction curve can be replaced by a broken line. In this case, it is sufficient to indicate the coordinates of a few characteristic points of the interaction curve.

### 4.1. Interaction n–m with Compression Bars

This section presents an analytical model for predicting the bearing capacity of rectangular concrete columns reinforced with any FRP bars. The use of force equilibrium and strain compatibility principles provides the most direct approach to analysis.

**Figure 2:**
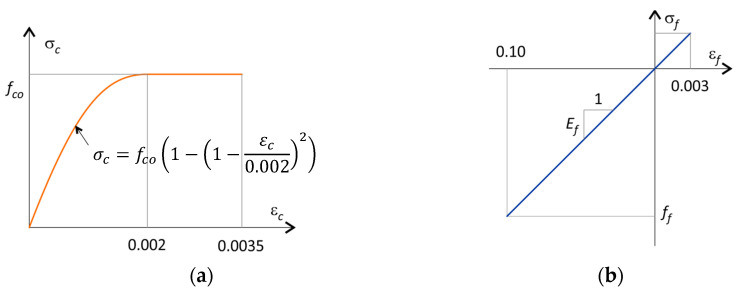
Material models for (**a**) concrete and (**b**) FRP bars.

The following assumptions were made in the analysis: (a) the plane sections remain plane, (b) the concrete strength is neglected, (c) a parabolic–rectangular stress diagram in the compression zone, (d) a linear elastic stress diagram for the tension and compression bars. Figure 2 shows the material models for concrete and FRP bars. Above this, the strength factors for concrete and FRP bars are set to unity, and the environmental reduction factor for FRP was also set to unity.

**Figure 3:**
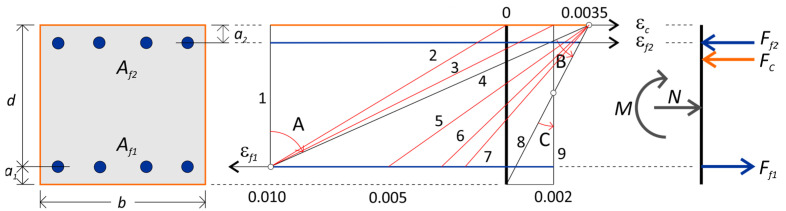
Cross section, strain diagrams and internal forces.

Figure 3 shows the strain profiles in the ultimate load state of a RC section from axial tension (strain profile 1) to eccentric tension (strain profile 2–4) and eccentric compression (strain profile 4–8) to axial compression (strain profile 9). The strength of the tensile reinforcement is determined by its limit strain ε_fu_ and the strength of the concrete by the limit strain ε_cu_. According to recommendations [34], the strain in FRP tensile bars is limited to ε_fu_ = 0.01.

According to EC2 [9], the limiting strain in the extremity of the concrete compression fibre ε_cu_ = 0.0035 is assumed to be no greater than 0.002 for axial compression. In the same range, the strain in the compression bars ε_f2_ change.

Lines from 1 to 9 in Figure 3 correspond to the nine strain states of the RC section considered, for which the formulae for the coordinates of the points on the interaction curve in the axis system of the normalised axial force n and bending moment m have been determined:

(3) $$n=\frac{N}{f_{co}bd}$$

(4) $$m=\frac{M}{f_{co}bd^{2}}$$

where: f_co_—compressive strength of concrete, b—width of section and d—effective height of the section.

The section is symmetrically reinforced (A_f1_ = A_f2_, a_1_ = a_2_). The cross-section is described by two parameters: the mechanical reinforcement ratio ω [10] and the position of the reinforcement in the section expressed by the factor β:

(5) $$\omega =\frac{A_{f1}}{bd}\frac{\varepsilon_{fu}E_{f}}{f_{co}}$$

(6) $$\beta =\frac{a_{1}}{d}$$

where: E_f_ is the modulus of elasticity of the FRP bars.

**Figure 4:**
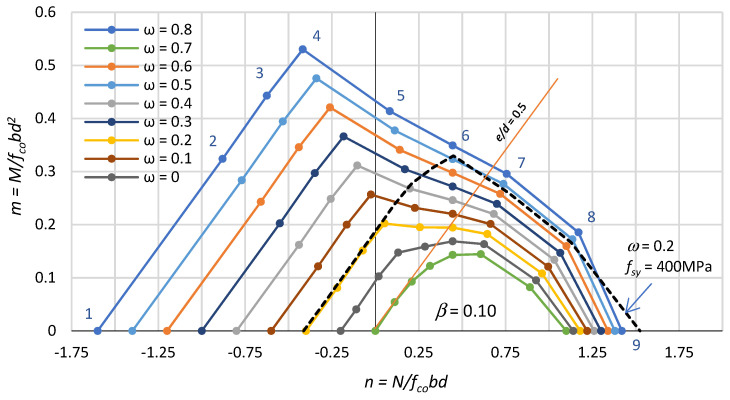
Effect of reinforcement intensity ω on the interaction n–m.

Based on the equilibrium of the cross-sectional forces, formulas were derived for calculating the coordinates of the nine points on the interaction curve corresponding to the nine strains profiles presented in Figure 3. These formulae are given in Table 1. Table 1 also gives the values of the relative compression zone depth ξ = x/d, where x is the compression zone depth. Figure 4 shows the graphs of the n–m relationship, developed on the basis of the formulae in Table 1, for different mechanical reinforcement ratios ω = 0–0.8, and a fixed reinforcement position, i.e., β = 0.10.

The fourth point on the interaction line (n_4_; m_4_) is the point at which the strains in the concrete and reinforcement simultaneously reach limit values, i.e., in the tension bars at 0.01 and in the extreme compression fibre of the concrete at 0.0035. Then, the height of the compression zone is equal to ξ = 7/27. This means that for a height of the compression zone ξ < 7/27, the tensile reinforcement decides the section load capacity (tension failure), and for a height of the compression zone ξ > 7/27, the concrete in the compression zone decides the section load capacity (compression failure). Point no. 4 is referred to as the balance failure point. The balance failure point for sections with FRP reinforcement takes place for eccentric tension, which is different from that for sections with steel reinforcement. In Figure 4, it can be seen that for a mechanical reinforcement ratio ω ≥ 0.3, the crushing of the concrete in the compressed zone (ε_cu_ = 0.0035) already takes place for eccentric tension.

The diagrams in Figure 4 show that for a fixed position of the reinforcement β, the bearing capacity of an eccentrically loaded section increases proportionally with the mechanical reinforcement ratio ω.

In practice, only the part of the diagram for eccentric compression is important, because the axial compressive force and bending moment most often act on reinforced columns. Cases where columns are tensioned eccentrically are encountered occasionally. Therefore, we most often have cases of calculating the required reinforcement for eccentrically compressed columns.

It can be seen that for eccentric compression, the RC section resistance depends on the eccentricity of the axial force. In Figure 4, the dashed line shows the interaction of n–m forces for a section reinforced with steel bars with yield strength f_sy_ = 400 MPa, E_s_ = 200 GPa and ω = 0.2. Comparing, in Figure 4, the interaction curves of sections with FRP and steel bars for ω = 0.2, it can be seen that their behaviour is the same during eccentric tension. However, for eccentric compression, the load capacity of sections with steel reinforcement is much higher. This is the result of a much higher ratio of steel reinforcement ρ for a constant mechanical reinforcement ratio ω. For a constant ratio reinforcement ρ, the load capacity of columns with steel reinforcement is lower.

**Figure 5:**
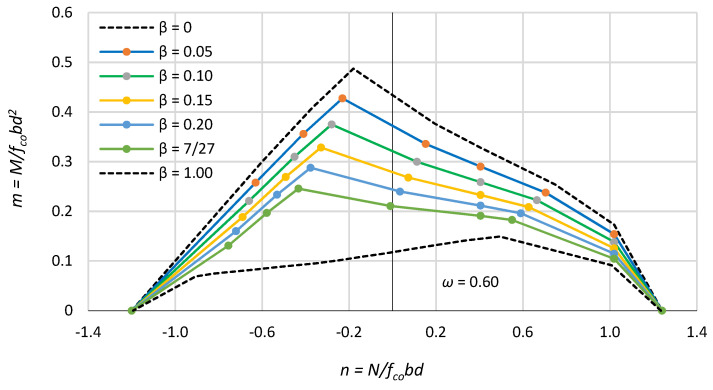
Effect of the location of the bars β in the section on the interaction n–m.

For a given mechanical reinforcement ratio ω, the location of the reinforcement in the section determines the resistance of the RC section. Figure 5 illustrates the effect of the location of the reinforcement in the RC section expressed by the coefficient β for a mechanical reinforcement ratio ω = 0.6 on the interaction of n–m forces.

As expected, placing the reinforcement closer to the neutral axis results in a reduction in the bearing capacity for both eccentric compression and eccentric tension. As the eccentricity of the axial force e/d approaches zero, the differences in RC section bearing capacity become smaller and smaller, and for axial compression, the differences disappear. Figure 5 also shows the n–m interaction lines for the two extreme cases of the position of the reinforcement, i.e., at the edges of the section (β = 0) and in the neutral axis of the section (β = 1.0).

The location of the bars in reinforced concrete columns depends primarily on the required cover thickness and varies in most cases within a β-factor of 0.10 to 0.20. In columns with FRP reinforcement, bars may be placed closer to the outer edges due to the lack of a need to protect this reinforcement from corrosion, the high strength of FRP bars and the smaller bar diameters used.

#### 4.1.1. The Method Validation

**Figure 6:**
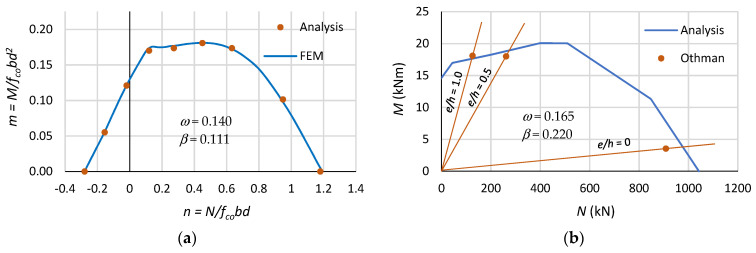
Comparisons: (**a**) analysis vs. FEM and (**b**) analysis vs. experimental results (data from [21]).

In order to validate the presented method, the results obtained from the proposed formulae were compared with the results obtained from FEM analyses performed with the XTRACT application from Imbsen Software Systems [40]. Figure 6a shows that the analytical calculation results coincide with the FEM analysis results. In addition, Figure 6b shows a comparison between the analytical results and the experimental results [21], in which rectangular columns reinforced with CFRP bars loaded axially and eccentrically were tested. Section dimensions: 150 mm × 150 mm, length column: 900 mm, longitudinal reinforcement: 4 ϕ 12 mm, CFRP (f_f_ = 2000 MPa, E_f_ = 150 GPa, ε_fu_ = 1.38%), concrete compressive strength: f_co_ = 44.7 MPa, axial force eccentricities: e/h = 0, 0.5 and 1.0. When the column’s (for e/h = 1.0) load capacity was reached, the measured deformation in the tension bars was equal to ε_f1_ ≅ 0.4%.

As can be seen in Figure 6b, the experimental results are similar to those obtained from the proposed analytical method. However, it can be seen that geometric imperfections had a large impact on the experimental results, and the axial compression (e/h = 0) is accompanied by a large bending moment. The convergence in the numerical analysis results and the experimental results with the analytical results allow us to conclude that the proposed method reasonably predicts the ultimate capacity of columns reinforced with FRP bars.

#### 4.1.2. Singularity of the Interaction Curve

The n–m interaction curve between points 4 and 8 (see Figure 4) is concave, which seems to be contrary to the generally known rules. According to plasticity theory, the convexity and normality of the limit surface does follow from Druckrer’s postulate for stable material [41].

According to the authors, this is a concavity of the n–m curve resulting from the adopted strain hypothesis of strength and the use of a linear elastic material of high strength-FRP bars and an elastic plastic material-concrete. The concavity of the curve can be proved by the existence of an inflection point on this curve.

The strain state of the section, for the pivot point B (Figure 3), is limited by the strain in the concrete ε_cu_ = 0.0035 and the section height ξ varying from 7/27 to (1 + β). The interaction curve equations in this strain range are as follows:

(7) $$m\left(\xi \right)=\frac{17}{27}\xi \left(\frac{1+\beta }{2}-\frac{99}{238}\xi \right)+\frac{7\omega }{40}\frac{{\left(1-\beta \right)}^{2}}{\xi }$$

(8) $$n\left(\xi \right)=\frac{17}{27}\xi +\frac{7\omega }{20}\left(2-\frac{1+\beta }{\xi }\right)$$

An inflection point on the curve exists when the second derivative of the parametric function is equal to zero:

(9) $$\frac{d^{2}m\left(\xi \right)}{dn^{2}\left(\xi \right)}=\frac{m\prime \prime \left(\xi \right)n\prime \left(\xi \right)-m\prime \left(\xi \right)n\prime \prime \left(\xi \right)}{{\left(n\prime \left(\xi \right)\right)}^{3}}=0$$

The solution of Equation (9) can be reduced to a third-degree polynomial:

(10) $$-\xi^{3}-\frac{441}{340}\omega \xi \left(1+\beta \right)+\frac{343}{330}\omega \left(1+\beta^{2}\right)=0$$

The analytical solution of Equation (10) is elaborate. As a result of the numerical solutions, the heights of the compressed zone ξo can be determined, and from this, the coordinates of the inflection point on the interaction curve (n(ξo); m(ξo)) can be calculated.

For example, for ω = 0.2 and β = 0.1, the height of the compression zone is ξo = 0.439, and the coordinates of the inflection point are n(0.439) = 0.320; m(0.439) = 0.195. The inflection point on the interaction curves occurs when ω ≥ 0.027. The concavity in the interaction curve is visible in Figure 6a for FEM analysis and has also been observed in other analyses of columns with hybrid reinforcement [26] and FRP reinforcement [26,28].

### 4.2. Interaction n–m without the Compression Bars

As mentioned earlier, the compressive strength of composite bars is much lower than their tensile strength [14]. Therefore, due to the orthotropic properties of FRP bars and the lack of sufficient experimental investigations, some standards [3,4] prescribe the neglect of compression FRP bars in the analysis of the section bearing capacity. Table 2 gives the formulae for the coordinates of the points of the n–m interaction curve for a symmetrically reinforced section without compression bars.

**Figure 7:**
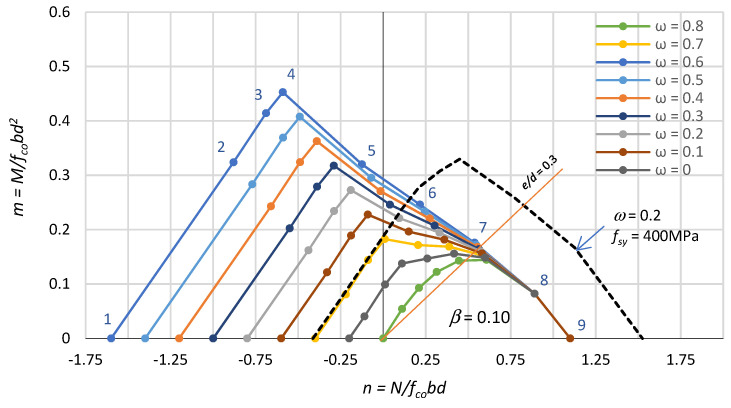
Effect of the mechanical reinforcement ratio ω on the interaction n–m without the compression bars.

Figure 7 shows the n–m interaction diagrams described by the coordinates of the points in Table 2 for a mechanical reinforcement ratio ω varying between 0.0 and 0.8. From these diagrams, it can be seen that the bearing capacity of eccentrically compressed sections in which compression reinforcement is neglected is significantly lower than when this reinforcement is included (see Figure 3). The bearing capacity differences found are small for eccentric tension, but for eccentric compression, they are very large. Figure 7 also shows, with a dashed line, the n–m interaction for a section reinforced with steel bars (f_sy_ = 400 MPa, ω = 0.2).

If the eccentricity of the axial force is e/d < 0.3, then the amount of reinforcement has no effect on the bearing capacity of the eccentrically compressed section. The neglect of the forces transmitted by the compression bars in the analysis of the bearing capacity of an eccentrically compressed section makes the use of FRP reinforcement in ‘heavily’ loaded columns problematic.

## 5. Required Reinforcement

**Figure 8:**
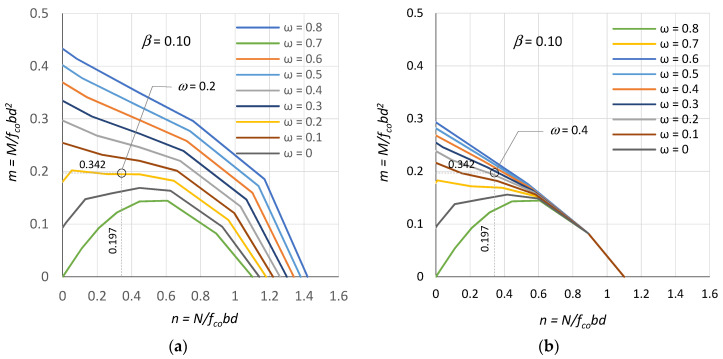
Curves of n–m interaction for sections: (**a**) with compression bars and (**b**) without compression bars.

Figure 8 shows the n–m interaction curves limited to eccentrically compressed RC sections for two cases: including the compression bar resistance (Figure 8a) and neglecting the compression bar resistance (Figure 8b). These are curves for a reinforcement position defined by a factor β = 0.10 and a mechanical reinforcement ratio varying from ω = 0 to ω = 0.8.

After determining the section dimensions (b, h), the position of the reinforcement in the section (a_1_) and the choice of concrete class (f_co_) have been determined, the values of the sectional forces (n, m) from the actions can be plotted on the nomograms, as shown in Figure 8, and the required mechanical reinforcement ratio (ω) can be read off. Then, on the basis of the determined mechanical reinforcement ratio ω and the selected type of RFP reinforcement (E_f_), the cross-sectional area of reinforcement required in the section can be calculated using the formula:

(11) $$A_{f1}=A_{f2}=bd\omega \frac{f_{co}}{\varepsilon_{fu}E_{f}}$$

**Figure 9:**
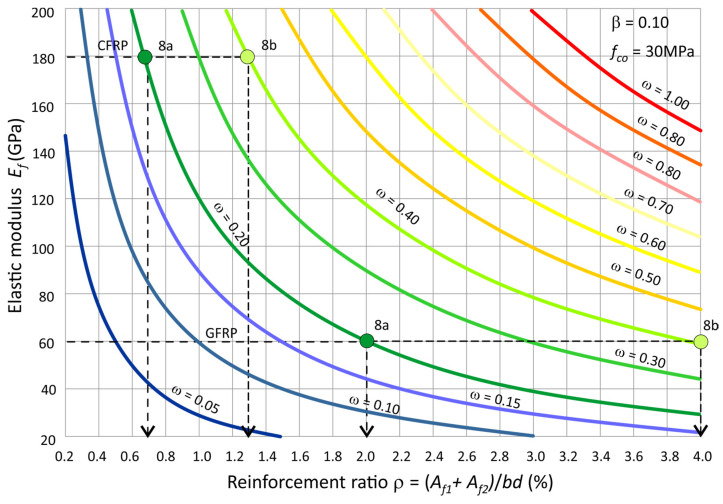
Nomogram for determining the required of reinforcement ratio ρ (β = 0.10, f_co_ = 30 MPa).

Figure 9 shows the diagrams of the mechanical reinforcement ratio ω as a function of the ratio of reinforcement ρ and the type of FRP bars defined by their elastic modulus E_f_. The diagrams were constructed for FRP bars with different fibre (E_f_ = 20–200 GPa) and reinforcement ratio ρ from minimum (0.2%) to maximum (4%), as recommended by EC2 [9]. These charts can be used as a nomogram to calculate the required reinforcement ratio (ρ) of the section based on the mechanical reinforcement ratio (ω) and the type of FRP bars (E_f_). Then, using the readout of the reinforcement ratio ρ from the nomogram, the cross-sectional area of the reinforcement A_f1_ and A_f2_ can be determined using the formula:

(12) $$\left(A_{f1}+A_{f2}\right)=\rho bd$$

### Application Example

We design a reinforcement with CFRP bars (E_f_ = 180 GPa) and GFRP bars (E_f_ = 60 GPa) in a column with cross-sectional dimensions h = 40 cm, b = 40 cm, a_1_ = 3.5 cm, d = 36.5 cm made of concrete with a compressive strength of f_co_ = 30 MPa for a combination of action effects (including or neglecting geometric imperfections and second order effects) where N = 1500 kN and M = 315 kNm (e = M/N = 21 cm). The calculation should be carried out for two cases: with and without the presence of compression bars.

For this purpose, the normalised bending moment m and axial force n from the considered load combination (N, M) is found using Equations (3) and (4). Then, the mechanical reinforcement ratio ω is read from Figure 8a,b, on this basis, the ratio of reinforcement ρ is read off from Figure 9, and the required reinforcement (A_f1_ + A_f2_) is calculated from (12). The results of the calculations are given in Table 3. In the example presented here, the characteristic strength values of concrete and FRP bars and loads are used.

## 6. Final Remarks

Non-metallic reinforcement made of FRP bars based on various types of fibre, usually glass and carbon fibre, is increasingly used in concrete structures. FRP reinforcement is used primarily in structures exposed to unfavourable environmental conditions. At present, only a few standards and recommendations allow the design of structures with this type of reinforcement. Numerous experimental studies are still being carried out and calculation methods are being developed with the aim of introducing rules for the design of concrete structures with FRP reinforcement into standards, including Eurocode 2 [9].

The behaviour of RC sections with FRP reinforcement is different from sections with steel reinforcement. Sections reinforced with FRP bars have a significantly higher eccentric tension carrying capacity than eccentric compression. The balance failure point for sections with FRP reinforcement takes place for eccentric tension. The interaction curves for the sections with FRP bars are concave at a certain segment.

In the design of reinforced concrete columns, axial force–bending moment interaction curves are very effective, as they allow the most unfavourable combination of loads and bearing capacity reserves to be easily determined. This paper presents n–m interaction curves for eccentrically loaded sections with FRP reinforcement based on current guidelines and standard recommendations. Interaction curves were developed for two cases: with and without compression reinforcement bars. The bearing capacity of sections where compression bars are not included is considerably lower especially for compression at low eccentricity since only the concrete part of the section carries the axial force. In this case, the FRP reinforcement only acts as a minimum structural reinforcement due to cracking, as the section’s load capacity is determined only by the concrete. Therefore, further experimental research and analysis are needed on the effect of FRP bars in compression on the load carrying capacity of concrete columns.

The bearing capacity of sections with FRP reinforcement depends on two parameters: the mechanical reinforcement ratio ω and the position of the reinforcement in the section expressed by the coefficient β.

The proposed method of designing the reinforcement is universal. Based on this procedure, any of the possible types of FRP bars and others with linear elastic characteristics can be designed. This procedure can be used in the design of column reinforcement, but then the design values of loads and material strengths considering partial safety factors and environmental reduction factor have to be introduced.

The analysis of the reinforced concrete section follows the provisions of the strength design method and the unified design rules, with all strength conditions satisfying the applicable equilibrium and deformation compatibility conditions. The presented analytical method and the developed nomogram allow a rational choice for the type of FRP bars and their amount in the section.

## Figures and Tables

**Table 1:** The coordinates of the points on the interaction curve n–m.

Strain Profile Number	$\xi =\frac{x}{d}$	$n_{i}=\frac{N_{i}}{f_{co}bd}$	$m_{i}=\frac{M_{i}}{f_{co}bd^{2}}$
1	-ꚙ	$n_{1}=-2\omega$	*m*_1_ = 0
2	0	$n_{2}=-\omega \left(1+\beta \right)$	$m_{2}=-\omega \frac{{\left(1-\beta \right)}^{2}}{2}$
3	1/6	$n_{3}=\frac{1}{9}-\frac{2}{5}\omega \left(2+3\beta \right)$	$m_{3}=\frac{1}{9}\left(\frac{1+\beta }{2}-\frac{1}{16}\right)+\frac{3}{5}\omega {\left(1-\beta \right)}^{2}$
4	7/27	$n_{4}=\frac{17}{81}-\frac{\omega }{20}\left(13+27\beta \right)$	$m_{4}=\frac{17}{81}\left(\frac{1+\beta }{2}-\frac{11}{102}\right)+\frac{27}{40}\omega {\left(1-\beta \right)}^{2}$
5	7/17	$n_{5}=\frac{17}{54}-\frac{\omega }{10}\left(2+9\beta \right)$	$m_{5}=\frac{161+238\beta }{1512}+\frac{18}{40}\omega {\left(1-\beta \right)}^{2}$
6	$\frac{1+\beta }{2}$	$n_{6}=\frac{17}{42}\left(1+\beta \right)$	$m_{6}=\frac{139}{1176}{\left(1+\beta \right)}^{2}+\frac{7}{20}\omega \frac{{\left(1-\beta \right)}^{2}}{1+\beta }$
7	3/4	$n_{7}=\frac{17}{28}+\frac{7}{15}\left(\frac{1}{2}-\beta \right)\omega$	$m_{7}=\frac{17}{28}\left(\frac{1+\beta }{2}-\frac{297}{952}\right)+\frac{7}{30}\omega {\left(1-\beta \right)}^{2}$
8	1+β	$n_{8}=\frac{17}{21}\left(1+\beta \right)+\frac{7}{20}\omega$	$m_{8}=\frac{10}{147}{\left(1+\beta \right)}^{2}+\frac{7}{40}\omega \frac{{\left(1-\beta \right)}^{2}}{1+\beta }$
9	+ꚙ	$n_{9}=1+\beta +\frac{4}{10}\omega$	$m_{9}=0$

**Table 2:** Coordinates of the interaction points of the n–m forces without compression bars.

Strain Profile Number	$\xi =\frac{x}{d}$	$n_{i}=\frac{N_{i}}{f_{co}bd}$	$m_{i}=\frac{M_{i}}{f_{co}bd^{2}}$
1	-ꚙ	$n_{1}=-2\omega$	$m_{1}=0$
2	0	$n_{2}=-\omega \left(1+\beta \right)$	$m_{2}=\omega \frac{{\left(1-\beta \right)}^{2}}{2}$
3	1/6	$n_{3}=\frac{1}{9}-\omega$	$m_{3}=\frac{1}{9}\left(\frac{1+\beta }{2}-\frac{1}{16}\right)+\omega \frac{1-\beta }{2}$
4	7/27	$n_{4}=\frac{17}{81}-\omega$	$m_{4}=\frac{17}{81}\left(\frac{1+\beta }{2}-\frac{11}{102}\right)+\omega \frac{1-\beta }{2}$
5	7/17	$n_{5}=\frac{17}{54}-\frac{11}{20}\omega$	$m_{5}=\frac{23+34\beta }{216}+\omega \frac{11}{20}\frac{{\left(1-\beta \right)}^{2}}{1+\beta }$
6	$\frac{1+\beta }{2}$	$n_{6}=\frac{17}{42}\left(1+\beta \right)-\omega \frac{7}{20}\frac{1-\beta }{1+\beta }$	$m_{6}=\frac{139}{1176}{\left(1+\beta \right)}^{2}+\omega \frac{7}{40}\frac{{\left(1+\beta \right)}^{2}}{1+\beta }$
7	3/4	$n_{7}=\frac{17}{28}-\omega \frac{7}{80}$	$m_{7}=\frac{17}{28}\left(\frac{1+\beta }{2}-\frac{297}{952}\right)+\omega \frac{7}{80}\frac{1-\beta }{2}$
8	1+β	$n_{8}=\frac{17}{21}\left(1+\beta \right)$	$m_{8}=\frac{10}{147}{\left(1+\beta \right)}^{2}$
9	+ꚙ	$n_{9}=1+\beta$	$m_{9}=0$

**Table 3:** Calculation results.

Bar Type	GFRP		CFRP	
Compression Bars	Yes	No	Yes	No
n, m from (3) (4)	n = 0.342, m = 0.197			
ω from (Figure 8a,b)	0.200	0.400	0.200	0.400
ρ from (Figure 9)	2.0%	4.0%	0.7%	1.3%
(A_f1_ + A_f2_) required from (12)	29.2 cm^2^	58.4 cm^2^	10.2 cm^2^	18.9 cm^2^
(A_f1_ + A_f2_) provided	2 × 5 ϕ 20 31.4 cm^2^	2 × 10 ϕ 20 62.8 cm^2^	2 × 3 ϕ 16 12.0 cm^2^	2 × 3 ϕ 20 18.8 cm^2^

## Data Availability

The data presented in this study are available on request from the corresponding author.

## Notation

The following symbols are used in this paper:

*A_f_*_1_, *A_f_*_2_	cross-sectional area of FRP reinforcement
*E_f_*	tensil elastic modulus of FRP reinforcement
*E_s_*	tensil elastic modulus of steel reinforcement
*M*, *M_u_*	bending moment, ultimate bending moment
*N*, *N_u_*	axial force, ultimate axial force
*a*_1_, *a*_2_	position in section of FRP reinforcement
*b*	section width
*d*	effective section height
*e*	eccentricity of axial force
*h*	section height
*f_co_*	compressive strength of concrete
*f_f_*	tensile strength of FRP reinforcement
*f_sy_*	yield strength of steel reinforcement
*n*, *m*	normalised axial force, normalised bending moment
*β*	position of the reinforcement in the section factor
*ξ*	relative compression zone depth
*ρ*	reinforcement ratio
*ε_cu_*	ultimate strain of concrete
*ε_fu_*	ultimate tensile strain of FRP longitudinal reinforcement
*ω*	mechanical reinforcement ratio

## References

[1] (2007). FRP Reinforcement in RC Structures.

[2] (2003). Guide for the Design and Construction of Concrete Reinforced with FRB Bars. ACI Committe 440.

[3] (2014). Building Code Requirements for Structural Concrete and Commentary.

[4] (2012). Design and Construction of Building Components with Fiber-Reinforced Polymers.

[5] (2006). Guide for the Design and Construction of Concrete Structures Reinforced with Fiber-Reinforced Polymer Bars.

[6] Japan Society of Civil Engineers (1997). Recommendation for design and construction of concrete structures using continuums fiber reinforcing materials. Concr. Eng. Ser. JSCE.

[7] Gudonis E., Timinskas E., Gribniak V., Kaklauskas A., Arnautov A.K., Tamulenas G. (2013). FRP reinforcement for concrete structures: State-of-the-art review of application and design. Eng. Struct. Technol..

[8] Kotynia R. Wymiarowanie i kształtowanie wybranych konstrukcji betonowych ze zbrojeniem niemetalicznym (Design and details of selected concrete structures with non-metallic reinforcement). Proceedings of the XXXIII Ogólnopolskie Warsztaty Pracy Projektanta Konstrukcji.

[9] (2004). Eurocode 2: Design of Concrete Structures—Part 1-1: General Rules and Rules for Buildings.

[10] Ye Y.-Y., Zhuge Y., Smith S.T., Zeng J.-J., Bai Y.-L. (2022). Behavior of GFRP-RC columns under axial compression: Assessment of existing models and a new axial load-strain model. J. Build. Eng..

[11] Ali S., Ahmad JSheikh M.N., Yu T., Hadi M.N.S. (2021). Analytical load-moment (P-M) interaction diagrams of GFRP bar reinforced circular geopolymer concrete columns. Structures.

[12] (2013). fib Model Code for Concrete Structures 2010.

[13] Szmigiera E.D., Protchenko K., Urbański M., Garbacz A. (2019). Mechanical properties of hybrid FRP bars and nano-hybrid FRP bars. Arch. Civ. Eng..

[14] AlNajmi L., Abed F. (2020). Evaluation of FRP bars under compression and their performance in RC columns. Materials.

[15] Khorramian K., Sadeghian P. (2021). Material characterization of GFRP bars in compression using a new test method. J. Test. Eval..

[16] Banibayat P., Patnaik A. (2015). Creep rupture performance of basalt fiber-reinforced polymer bars. J. Aerosp. Eng..

[17] Solyom S., Balazs G.L. (2020). Bond of FRP bars with different surface characteristics. Constr. Build. Mater..

[18] Ascione L., Gutierez E., Dimova S., Pinto A., Denton S. (2016). Prospect for New Guidance in the Design of FRP..

[19] Afifi M.Z., Mohamed H.M., Benmokrane B. (2014). Strength and Axial Behavior of Circular Concrete Columns Reinforced with CFRP Bars and Spirals. J. Compos. Constr..

[20] Hadhood A., Mohamed H.M., Benmokrane B. (2017). Axial load–moment interaction diagram of circular concrete columns reinforced with CFRP bars and spirals: Experimental and theoretical. J. Compos. Constr..

[21] Othman Z.S., Mohammad A.H. (2019). Behaviour of eccentric concrete columns reinforced with carbon fibre-reinforced polymer bars. Adv. Civ. Eng..

[22] Tobbi H., Farghaly A.S., Benmokrane B. (2014). Behavior of concentrically loaded fiber-reinforced polymer reinforced concrete columns with varying reinforcement types and ratios. ACI Struct. J..

[23] Tobbi H., Farghaly A.S., Benmokrane B. (2012). Strength model for concrete columns reinforced with fiber-reinforced polymer bard and ties. ACI Struct. J..

[24] Issa M.S., Metwally I.M., Elzeiny S.M. (2011). Structural performance of eccentrically loaded GFRP reinforced concrete columns. Int. J. Civ. Struct. Eng..

[25] Bakouregiu A.S., Mohamed H.M., Yahia A., Benmokrane B. (2021). Axial load–moment interaction diagram of full-scale circular LWSCC columns reinforced with BFRP and GFRP bars and spirals: Experimental and theoretical investigations. Eng. Struct..

[26] AlHamaydeh M., Amin F. (2021). Data for interaction diagrams of geopolymer for slender columns with double-layer GFRP and steel reinforcement. Data.

[27] Korentz J. (2022). Nośność mimośrodowo ściskanych słupów betonowych ze zbrojeniem niemetalicznym (Bearing capacity of eccentrically compressed concrete columns with non-metallic reinforcement). Builder.

[28] Isleem H.F., Tayeh B.A., Alaloul W.S., Musarat M.A., Raza A. (2021). Artificial neural network (ANN) and finite element (FEM) models for GFRP-reinforced concrete columns under axial compression. Materials.

[29] Youssef J., Hadi M.N.S. (2017). Axial load-bending moment diagrams of GFRP reinforced columns and GFRP encased square columns. Constr. Build. Mater..

[30] Korentz J. (2020). Influence of geometric imperfections on buckling resistance of reinforcing bars during inelastic deformation. Materials.

[31] Hales T.A., Pantelides C.P., Reaveley L.D. (2016). Experimental evaluation of slender high-strength concrete columns with GFRP and hybrid reinforcement. J. Compos. Constr..

[32] Won J.P., Park C.G., Kim H.H., Lee S.W., Jang C.I. (2008). Effect of fibers on the bonds between FRP reinforcing bars and high-strength concrete. Compos. Part B Eng..

[33] Raza A., Khan Q.Z. (2020). Experimental and Theoretical Study of GFRP hoops and spirals in hybrid fiber reinforced concrete short columns. Mater. Struct..

[34] Zadeh H.J., Nanni A. (2013). Design of RC columns using glass FRP reinforcement. J. Compos. Constr..

[35] Hadi M.N.S., Karim H., Sheikh M.N. (2016). Experimental investigations on circular concrete columns reinforced with GFRP bars and helices under different loading conditions. J. Compos. Constr..

[36] De Luca A., Matta F., Nanni A. (2010). Behavior of full-scale glass fiber-reinforced polymer reinforced concrete columns under axial loading. ACI Struct. J..

[37] Dogan G., Arslan M.H. (2016). Failure modes of RC columns under loading. Int. J. Sci. Eng. Res..

[38] Maekawa K., An X. (2000). Shear failure and ductility of RC columns after yielding of main reinforcement. Eng. Fract. Mech..

[39] Mari A.R., Hellesland J. (2005). Lower slenderness limits for rectangular reinforced concrete columns. J. Struct. Eng. ASCE.

[40] https://softadvice.informer.com/Imbsen_Software_Systems.html.

[41] Chen W.-F., Atsuta T. (1977). Theory of Beam Column. Volume 1: In-Plane Behaviour and Design.

